# Enlargements of the Spleen—VI

**Published:** 1908-09-12

**Authors:** 


### MEDICINE.

### ENLARGEMENTS OF THE SPLEEN—VI.

### SPLENIC ENLARGEMENTS WITH DISTINCTIVE BLOOD CHANGES (*continued.*)

ONE may divide cases of splenic tumour into two main groups, namely:—

I. Those cases in which the blood changes are positive and distinctive.

II. Those cases in which the blood changes are negative, or not distinctive.

Group I. includes spleno-medullary leuchæmia, lymphatic leuchæmia, pernicious anæmia, malaria, spleno-megalic polycythæmia, and typhoid fever. Group II. includes all the remainder. The two groups may be discussed separately.

#### I. SPLENIC ENLARGEMENTS WITH DISTINCTIVE BLOOD CHANGES.

(1) *Spleno-medullary Leuchæmia.* — Spleno-medullary leuchæmia gives rise to the largest of all spleens, weighing anything between 2 and 20 lb., and sometimes filling three-quarters of the abdomen. There must be a period in the disease when the spleen is less big than this, however, so that the mere size of the organ will not make the diagnosis certain one way or the other. A correct diagnosis is afforded at once by the blood counts, which are pathognomonic. The characteristic changes are in the white corpuscles, which are not only enormously increased in total numbers—up to something between 100,000 and 600,000 per cub. mm., but also exhibit a very distinctive differential count in that neutrophile and eosinophile myelocytes are present to the extent of between 20 per cent. and 60 per cent. of all the leucocytes in the blood. The red cells are not appreciably altered in the early stages; later they become more and more diminished; the hæmoglobin falls still more rapidly, so that the colour index becomes lower and lower; poikilocytes and nucleated red corpuscles become a marked feature of the films in late stages. None of these changes are essential, however; whereas, if with a big spleen, there are more than 50,000 leucocytes per cub. mm., and of these a considerable proportion are myelocytes, the diagnosis cannot be anything else than spleno-medullary leuchæmia.

(2) *Lymphatic Leuchæmia.*—In lymphatic leuchæmia the spleen may be only slightly or moderately enlarged, and it is very seldom as big as it is in a decided case of spleno-medullary leuchæmia. It is, moreover, softer than it is in the latter disease. The superficial lymphatic glands are usually enlarged, there is a marked tendency to purpura and other hæmorrhages, the patient becomes markedly anæmic without much wasting, and the disease often runs an acute course ending fatally in three or four months or less. In some instances the lachrymal and salivary glands are appreciably swollen in such a way as to suggest mumps at the first onset.

The diagnosis can only be made with certainty by examining the blood. The latter is paler than usual; the changes in the red corpuscles are not pathognomonic, being precisely similar to those already described for spleno-medullary leuchæmia. The changes in the white corpuscles, on the other hand, are absolutely distinctive. There is an absolute increase in their numbers, up to anything between 50,000 and 500,000 per cub. mm. The leucocytic increase is generally less than it is in the spleno-medullary cases, the average in Professor Osler's nine cases being 144,800 per cub. mm. The differential leucocyte count at once distinguishes this form of leuchæmia from the other, for myelocytes are very few and far between, whereas the mononuclear lymphocytes constitute as many as from 80 to 90 per cent., and in some cases 95 or 97 per cent., of all the leucocytes present. This lymphocythæmia is highly characteristic. The cells may be either of the small or of the large type; upon the whole the more acute the disease the more likely are the large mononuclear cells to preponderate.

(3) *Mixed Leuchæmia.*—Although most cases of leuchæmia can be at once placed either in the spleno-medullary or in the lymphatic groups, it occasionally happens that the blood shows some of the characters of each type. The spleen may be considerably enlarged, the lymphatic glands may or may not be affected, there will be a marked leucocytosis, and the differential leucocyte count will show both considerable numbers of myelocytes and a high percentage of lymphocytes. In all leuchæmias there is leucocytosis, but it is the differential leucocyte count which decides the type of the leuchæmia; if myelocytes are numerous and the other cells consequently show a relative diminution, the disease is termed spleno-medullary; if lymphocytes are very numerous and myelocytes almost absent, the disease is styled lymphatic; if lymphocytes and myelocytes are both relatively numerous the disease partakes of both types and it is termed mixed.

(4) *Pernicious Anæmia.*—It is usually stated that the spleen is not enlarged in pernicious anæmia. This, however, is a mistake, for although the increase in size is seldom great, the organ is distinctly palpable in nearly half the cases, just as is the liver. The appearance of the patient—the primrose-yellow tint of the skin without any jaundice of the conjunctivæ—may suggest the diagnosis at once when the condition has reached a late stage. Earlier the diagnosis may be quite difficult. In any case, the only way of clinching the diagnosis is by blood examination, the most pathognomonic point being the high colour index in the presence of marked oligocythæmia. Thus, if the red corpuscles are reduced to 1,000,000 per cub. mm., or 20 per cent. of normal, the hæmoglobin might only be reduced to 25 per cent of normal. The colour index would then be $\frac{25}{20}$, or 1.25. The presence of marked anæmia with a high colour index is pathognomonic of pernicious anæmia; but it must be added the converse is not absolutely true. If a pernicious anæmia patient improves under treatment, the red corpuscles increase more rapidly than does the hæmoglobin, so that the colour index becomes less than 1 as the patient becomes better, although the condition is still one of pernicious anæmia. The same probably applies to the earlier stages of the disease; before the anæmia becomes marked, there may be a stage during which the colour index is low, antecedent to the severer stage when the typical high colour index is to be found.

There is no leucocytosis in pernicious anæmia, but rather a leucopenia, 2,500 leucocytes per cub. mm. of blood being quite a common figure. The differential leucocyte count also becomes abnormal, but not in an absolutely pathognomonic way. The small lymphocytes become relatively more numerous, the polymorphonuclear cells diminish, there may be a slight increase in the coarsely granular eosinophile cells, and not infrequently a few basophile cells and myelocytes may be seen. A typical, but not pathognomonic, differential leucocyte count in pernicious anæmia would thus be as follows:—

| | per cent. |
|---|---|
| Small lymphocytes | 39 |
| Large lymphocytes | 2 |
| Polymorphonuclear cells | 54 |
| Coarsely granular eosinophile cells | 3 |
| Basophile cells | 1 |
| Myelocytes | 1 |

The red corpuscles in a marked case exhibit an extreme degree of poikilocytosis—that is to say variation in shape; great variations in size, with a preponderance of megalocytes; a marked tendency to punctate basophilia, also termed polychromatophilia, the affected red cells not staining entirely red in Jenner's stain, but presenting minute points of blue amid the red; and in severe cases a variable number of nucleated red cells of various sizes may be present.

The changes in the red cells, severe though they are, are not absolutely pathognomonic, although if a film exhibits scarcity of leucocytes, abundance of myelocytes, and considerable poikilocytosis, there will be a great probability that the disease is pernicious anæmia.

To be absolutely certain of the diagnosis, however, it is essential that the numbers of red and white corpuscles per cub. mm. of blood should be counted, and the hæmoglobin estimated, in order that the colour index may be calculated. The pathognomonic points about the blood in pernicious anæmia are: leucopenia, oligocythæmia, and a colour index greater than 1.

*(To be continued.)*

